# Relationship between vaginal group B streptococcus colonization in the early stage of pregnancy and preterm birth: a retrospective cohort study

**DOI:** 10.1186/s12884-021-03624-9

**Published:** 2021-02-16

**Authors:** Sho Tano, Takuji Ueno, Michinori Mayama, Takuma Yamada, Takehiko Takeda, Kaname Uno, Masato Yoshihara, Mayu Ukai, Teppei Suzuki, Yasuyuki Kishigami, Hidenori Oguchi

**Affiliations:** 1 Department of Obstetrics, Perinatal Medical Center, TOYOTA Memorial Hospital, 1-1 Heiwa-cho, Toyota, Aichi 471-8513 Japan; 2 Department of Obstetrics and Gynecology, Nagoya University Graduate School of Medicine, 65 Tsurumai-cho, Showa-ku, Nagoya, Aichi 466-8550 Japan

**Keywords:** Preterm birth, Group B Streptococcus, Vaginal flora

## Abstract

**Background:**

Although infection and inflammation within the genital tract during pregnancy is considered a major risk factor for spontaneous preterm birth (PTB), there are few studies on association between vaginal microorganisms in the early stage of pregnancy and PTB. The aim of this study was to investigate relationship between vaginal Group B streptococcus (GBS) colonization, a leading cause of infection during pregnancy, in the early stage of pregnancy and PTB.

**Methods:**

This single-center, retrospective cohort study utilized data from 2009 to 2017 obtained at TOYOTA Memorial Hospital. Women with singleton pregnancies who underwent vaginal culture around 14 weeks of gestation during their routine prenatal check-up were included. Vaginal sampling for Gram staining and culture was performed regardless of symptoms. GBS colonization was defined as positive for GBS latex agglutination assay. Statistical analysis was performed to determine the factors associated with PTB.

**Results:**

Overall 1079 singleton pregnancies were included. GBS (5.7%) and *Candida albicans* (5.5%) were the most frequently observed microorganisms. The incidence of PTB (before 34 and before 37 weeks of gestation) were significantly higher in the GBS-positive group than in the GBS-negative group (6.6% vs 0.5%, *p* = 0.001 and 9.8% vs 4.3%, *p* = 0.047). Our multivariable logistic regression analysis revealed that GBS colonization was a factor associated with PTB before 34 and before 37 weeks of gestation (Odds ratio [OR] 15.17; 95% confidence interval [CI] 3.73–61.74), and OR 2.42; 95%CI 1.01–5.91, respectively).

**Conclusions:**

The present study found that vaginal GBS colonization in the early stage of pregnancy was associated with PTB. Our study indicates that patients at a high risk for PTB can be extracted by a simple method using conventional culture method.

**Supplementary Information:**

The online version contains supplementary material available at 10.1186/s12884-021-03624-9.

## Background

The presence of infection and inflammation within the genital tract during pregnancy has been considered a major risk factor for spontaneous preterm birth (PTB) [1]. The primary colonizing bacteria in healthy pregnant women are *Lactobacillus*, which produce protection against pathogenic species [2]. Bacterial vaginosis (BV) diagnosed based on Nugent scoring system (Table S1) is an independent risk factor for PTB. The risk for PTB is reported to be higher if BV occurs in the early stage of pregnancy [3]. The Nugent scoring method quantifies the presence of *Lactobacillus* morphotypes compared to Gram negative organisms and Gram variable *Actinobacteria* [4].

Regarding vaginal microorganisms, Group B streptococcus (GBS) is a leading cause of infection during pregnancy, and vaginal GBS colonization is a risk factor for developing neonatal GBS disease [5]. Detecting GBS during the last 5 weeks before delivery is believed to be accurate in predicting the presence of GBS in the mother during childbirth [6]. It is well known that vertical transmission of this organism in babies can be prevented by administration of prophylactic antibiotics to mothers during labor, so GBS screening is usually carried out at 35–37 weeks of gestation [7]. Also, there is a higher possibility of developing maternal GBS related diseases such as urinary tract infection or bacteremia [8, 9], which is worth noting that maternal infection can lead to PTB. Despite the practical importance of vaginal GBS colonization, limited studies have discussed the association between PTB and vaginal GBS colonization in the early stage of pregnancy. Although vaginal GBS colonization happens transiently, intermittently, or chronically in pregnant women [10], pregnant women who have vaginal GBS colonization in the early stage of pregnancy can be more affected by GBS.

The present study aims to investigate the prevalence of abnormal vaginal microorganisms during pregnancy and the relationship between GBS colonization in the early stage of pregnancy and PTB.

## Methods

### Study population

A single-center, retrospective cohort study was conducted using electronic health records from 2009 to 2017 obtained from TOYOTA Memorial Hospital, a perinatal center in Toyota city, Aichi, Japan. Women with pregnancies who underwent vaginal culture in the early stage of pregnancy (around 14 weeks of gestation) during their routine prenatal check-up at our hospital were included, whereas those who had known risks for spontaneous PTB (previous PTB, uterine malformation, myoma uteri or multiple pregnancies) or could require PTB due to medical indication (preeclampsia, fetal growth restriction, fetal malformation or intra-uterine fetal demise) were excluded at analysis. The requirement for informed consent was waived by the ethics committee due to the retrospective nature of the study. The ethics committee of TOYOTA Memorial Hospital approved this study.

### Sample collection

Vaginal sampling for Gram staining and culture were performed in the early stage of pregnancy for all pregnant women, regardless of the symptoms. After the insertion of sterile speculum using water-based lubrication, a smear was taken from the vagina using a sterile cotton swab. The Gram stain-based Nugent score was calculated, and the entire specimen was incubated on Nissui Separated Sheep Blood Agar/Chocolate Agar EX II® (Nissui Pharmaceutical Co., Ltd., Japan) for 24 h at 35 °C in 5% CO_2_, according to the manufacturer’s instruction. Negative plates were re-incubated for an additional 24 h and then reexamined. Positive plates were tested using GBS latex agglutination assay, Strept LA® (Denka Seiken Co., Ltd., Japan). It was selected based on cost, availability, and ease of use. GBS colonization was defined as positive for Strept LA®, and the detection of GBS during pregnancy prompted the administration of ampicillin during labor.

### Factors assessed

In order to investigate the relationship between vaginal GBS detection in the early stage of pregnancy and PTB, patients were divided into the GBS-positive group and the GBS-negative group according to the result of vaginal culture in the early stage of pregnancy. Baseline maternal characteristics including maternal age and complications such as chronic hypertension, diabetes mellitus and thyroid diseases were collected, whereas the incidence of BV (Nugent score of ≥7 points) and the results of vaginal culture were examined. Nullipara was defined as having no previous history of a delivery after 20 weeks of gestation. Overweight is defined as a prepregnant body mass index (BMI) of 25 or more, and thyroid disease includes hyperthyroidism and hypothyroidism. Gestational diabetes mellitus (GDM) is diagnosed according to the oral glucose tolerance test (OGTT) international consensus criteria; the fasting plasma glucose level exceeds 92 mg/dL, the 1 h level exceeds 180 mg/dL or the 2 h level exceeds 153 mg/dL after 75 g glucose loading. Gestational age at delivery, birth weight, premature rupture of membranes (PROM) were defined as delivery outcomes. The preterm PROM (pPROM) is defined as the onset of amniotic fluid leakage from the vagina before the onset of uterine contractions at < 37 weeks of gestation [11], and the diagnosis of pPROM is based on both history and physical examination: visualization of pooling of amniotic fluid in the vaginal fornix or detecting insulin growth factor binding protein-1 (IGFBP-1) using check PROM® (Alfresa Pharma Co., Ltd., Japan). Statistical analysis was performed to determine the risk for PTB (earlier than 34 weeks and earlier than 37 weeks).

### Statistical analysis

Data are presented as means ± standard deviation or median [range] for continuous variables and n (%) for categorical variables. Baseline characteristics between GBS group and control group were compared using χ^2^ test, Student’s t-test or Mann–Whitney’s U test, as appropriate. Associations of individual parameters and PTB < 37 weeks of gestation or PTB < 34 weeks of gestation were calculated using univariable and multivariable regression analyses. Variables with *p*-values of less than 0.25 in the univariable regression analysis were entered to multivariable logistic regression analysis using backward elimination method. A *p*-value of < 0.05 was considered statistically significant. Statistical analyses were conducted using SPSS version 26.0 for Windows software (SPSS, Inc., Chicago, IL, USA).

## Results

### Participants

**Fig. 1:**
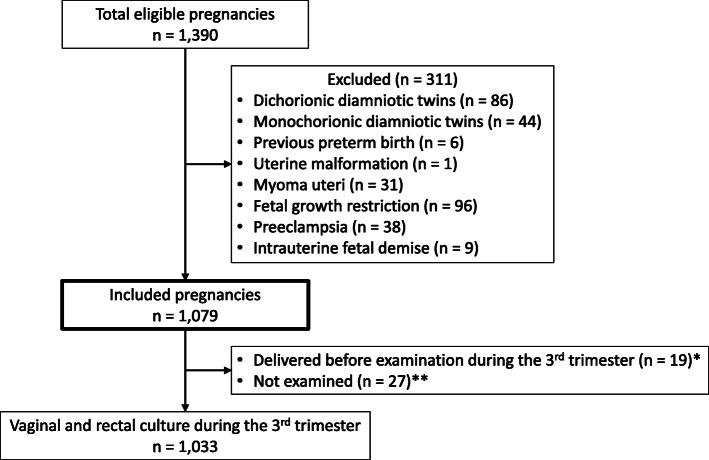
Flow chart describing subjects in this study. *Of the 19 patients, 3 were GBS-positive in early stage of pregnancy. **Of the 27 patients, 1 was GBS-positive in early stage of pregnancy

A total of 1390 pregnancies underwent vaginal culture examination in the early stage of pregnancy (around 14 weeks of gestation) at our institution during the study period. We excluded 311 patients because of known risks for spontaneous PTB (multiple pregnancy, previous PTB, uterine malformation or myoma uteri) or need for PTB due to medical indication (fetal growth restriction, preeclampsia or intra-uterine fetal demise; Fig. 1). The remaining 1079 patients were finally included.

### Vaginal flora

The prevalence of BV in the early stage of pregnancy was 6.9% (Table 1), with the most frequently observed microorganisms being GBS (5.7%) and *Candida albicans* (5.5%). The frequency of detecting GBS and *Candida albicans* from vaginal and rectal culture during the 3rd trimester as routine check-up was 9.5 and 7.2%, respectively (Table 1). The prevalence of BV in the early stage of pregnancy was lower in the GBS-positive group but there was no significant difference (3.3% vs 7.1%, *p* = 0.429; Table S2). Among 61 pregnant women who detected GBS in the early stage of pregnancy, 31 (50.1%) were also detected with GBS during the 3rd trimester. Of the remaining 30 patients, 26 were GBS-negative during the 3rd trimester, and 4 did not undergo vaginal and rectal cultures during the 3rd trimester. Of the 26 patients with GBS-negative during the 3rd trimester, 1 was complicated with GBS and symptomatic BV in the early stage of pregnancy, and was treated with metronidazole before the 3rd trimester.

**Table 1:** Prevalence of vaginal microorganisms in the early stage of pregnancy and during the 3rd trimester

	Early stage of pregnancy	3rd trimester
	Vagina *n* = 1079	Vagina and rectum *n* = 1033
GA at examination, wk. [range]	14.4 [11.3–17.7]	34.6 [33.1–36.9]
Nugent score		
0–3	888 (82.3)	903 (87.4)
4–6	117 (10.8)	75 (7.3)
7–10 (Bacterial vaginosis)	74 (6.9)	55 (5.3)
Abnormal bacterial colonization		
GBS		
Positive	61 (5.7)	98 (9.5)
Negative	1018 (94.3)	935 (90.5)
*Candida albicans*		
Positive	59 (5.5)	74 (7.2)
Negative	1020 (94.5)	959 (92.8)
*Candida glabrata*		
Positive	25 (2.3)	15 (1.5)
Negative	1054 (97.7)	1018 (98.5)
*Candida krusei*		
Positive	2 (0.2)	1 (0.1)
Negative	1077 (99.8)	1032 (99.9)
CNS		
Positive	18 (1.7)	107 (10.4)
Negative	1061 (98.3)	926 (89.6)
*Staphylococcus aureus*		
Positive	10 (0.9)	14 (1.4)
Negative	1069 (99.1)	1019 (98.6)
Enterococcus species		
Positive	7 (0.6)	20 (1.9)
Negative	1072 (99.4)	1013 (98.1)
Escherichia coli		
Positive	2 (0.2)	4 (0.4)
Negative	1077 (99.8)	1029 (99.6)
MRSA		
Positive	0 (0.0)	1 (0.1)
Negative	1079 (100)	1032 (99.9)
Others		
Positive	11 (1.0)	10 (1.0)
Negative	1068 (99.0)	1032 (99.0)

Data are presented as median [range] for continuous variables and n (%) for categorical variables

*GA* Gestational age, *GBS* Group B Streptococcus, *CNS* Coagulase-negative staphylococci, *MRSA* Methicillin-resistant Staphylococcus aureus

### Association between GBS colonization and pPROM

**Fig. 2:**
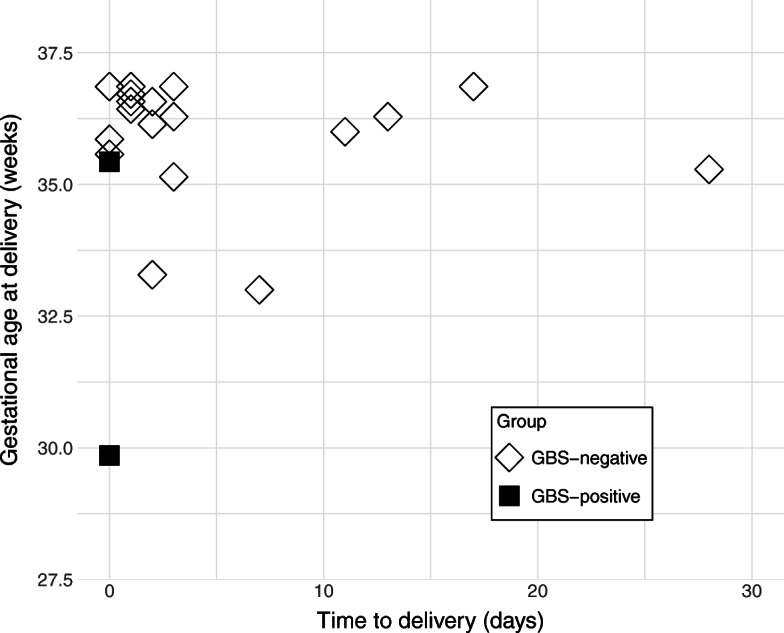
Time from the onset of preterm PROM to delivery. PROM, Premature rupture of membranes; GBS, Group B Streptococcus

As shown in Table 2, there were no significant differences in the baseline data including gestational age at vaginal culture, maternal age, and the incidence of diabetes mellitus and thyroid disease between GBS-positive and GBS-negative groups. The rate of nulliparity was significantly higher in the GBS-positive group (70.8% vs. 58.0%, *p* = 0.042), but there were no patients having episodes of cervical insufficiency among GBS-positive patients included. The incidence of pPROM and term PROM were higher in the GBS-positive group, but there were no significant differences (3.3% vs. 1.9%, *p* = 0.335 and 23.0% vs. 14.2%, *p* = 0.062, respectively). As shown in Fig. 2, all 21 pPROM patients delivered at < 37 weeks of gestation, and 3 of 4 patients with pPROM at < 34 weeks of gestation delivered at < 34 weeks of gestation. The time to delivery of the 4 patients with pPROM at < 34 weeks of gestation was 0, 2, 7, and 28 days, respectively, and the shortest one was GBS-positive in the early stage of pregnancy.

**Table 2:** Comparison of baseline characteristics among women divided according to vaginal flora in early stage of pregnancy

	GBS in early stage of pregnancy		
	Positive *n* = 61	Negative *n* = 1018	*p*-value
GA at examination, weeks, median [range]	14.3 [13.6–16.3]	14.4 [11.3–17.7]	0.863
Maternal age, years old	34.8 ± 4.6	34.2 ± 4.9	0.277
Nullipara			
Yes	43 (70.5)	594 (58.3)	0.042*
No	18 (29.5)	424 (41.7)	
Overweight (BMI ≥ 25)			
Yes	6 (9.8)	99 (9.7)	0.977
No	55 (90.2)	919 (90.3)	
Diabetes mellitus			
Yes	3 (4.9)	27 (2.7)	0.417
No	58 (95.1)	991 (97.3)	
Thyroid disease			
Yes	2 (3.3)	71 (7.0)	0.427
No	59 (96.7)	947 (93.0)	
GDM			
Yes	5 (8.2)	123 (12.1)	0.373
No	56 (91.8)	895 (87.9)	
Preterm PROM			
Yes	2 (3.3)	19 (1.9)	0.335
No	59 (96.7)	999 (98.1)	
Term PROM			
Yes	14 (23.0)	146 (14.3)	0.062
No	47 (77.0)	872 (85.7)	
GA at delivery, weeks, median [range]	39.4 [29.9–42.0]	39.4 [32.3–42.7]	0.938
Preterm birth < 34 weeks			
Yes	4 (6.6)	5 (0.5)	0.001*
No	57 (93.4)	1013 (99.5)	
Preterm birth < 37 weeks			
Yes	6 (9.8)	44 (4.3)	0.047*
No	55 (90.2)	974 (95.7)	
Sex			
Male	32 (52.5)	533 (52.4)	0.988
Female	29 (47.5)	485 (47.6)	
Birth weight, kg, median [range]	3.0 [1.4–4.4]	3.1 [1.5–4.3]	0.433
Apgar score at 1 min			
Low Apgar score (< 7 points)	6 (9.8)	53 (5.2)	0.122
≥ 7 points	55 (90.2)	965 (94.8)	
Apgar score at 5 min			
Low Apgar score (< 7 points)	3 (4.9)	13 (1.3)	0.057
≥ 7 points	58 (95.1)	1005 (98.7)	
NICU admission			
Yes	10 (16.4)	109 (10.7)	0.168
No	51 (83.6)	909 (89.3)	
Neonatal GBS disease			
Yes	0 (0.0)	0 (0.0)	–
No	61 (100)	1018 (100)	

Data are presented as means ± standard deviation or median [range] for continuous variables and n (%) for categorical variables

*GBS* Group B Streptococcus, *GA* Gestational age, *BMI* Body mass index, *GDM* Gestational diabetes mellitus, *PROM* Premature rupture of membranes, *NICU* Neonatal Intensive Care Unit

*Statistically significant

### Association between GBS colonization and PTB

Table 3 revealed GBS colonization in the early stage of pregnancy was associated with increased risk for PTB at < 37 weeks (9.8% vs. 4.3%, Odds Ratio [OR] 2.42; 95% confidence interval [CI] 1.01–5.91) and at < 34 weeks (6.6% vs. 0.5%, OR 14.22; 95%CI 3.72–54.38). Multivariable logistic regression analyses to identify factors associated with PTB < 37 weeks of gestation and one of PTB < 34 weeks of gestation are shown in Table 4. Variables with *p*-values of less than 0.25 in the bivariable regression analysis were entered to multivariable logistic regression analysis using backward elimination method, and the final set for identifying the risks of PTB < 37 weeks of gestation was only “GBS-positive in the early stage of pregnancy” (OR 2.42; 95%CI 1.01–5.91). In addition to GBS-positive in the early stage of pregnancy, diabetes mellitus was associated with increased odds of PTB < 34 weeks of gestation in the multivariable regression analysis (OR 15.17; 95% CI 3.73–61.74, and OR 8.06; 95%CI 1.36–47.78, respectively).

**Table 3:** Comparison of characteristics of patients divided according to preterm birth

Variable	Preterm birth < 34 weeks of gestation					Preterm birth < 37 weeks of gestation				
	Yes n (%)	No n (%)	OR	95%CI	*p*-value	Yes n (%)	No n (%)	OR	95%CI	*p*-value
Maternal age, years old			0.98	(0.86–1.12)	0.748			0.97	(0.91–1.02)	0.210
Nullipara										
Yes	3 (0.5)	634 (99.5)	0.34	(0.09–1.38)	0.133	25 (3.9)	612 (96.1)	0.68	(0.39–1.20)	0.186
No	6 (1.4)	336 (98.6)	Ref	Ref		25 (5.7)	417 (94.3)	Ref	Ref	
Overweight (BMI ≥ 25)										
Yes	0 (0.0)	105 (100)	–	–	–	2 (1.9)	103 (98.1)	0.375	(0.09–1.56)	0.178
No	9 (0.9)	965 (90.1)				48 (4.9)	926 (95.1)	Ref	Ref	
Diabetes mellitus										
Yes	2 (6.7)	28 (93.3)	10.63	(2.11–53.50)	0.004*	3 (10.0)	27 (90.0)	2.37	(0.69–8.09)	0.169
No	7 (0.7)	1042 (99.3)	Ref	Ref		47 (4.5)	1002 (95.5)	Ref	Ref	
Thyroid disease										
Yes	0 (0.0)	73 (100)	–	–	–	3 (4.1)	70 (95.9)	0.87	(0.27–2.88)	0.825
No	9 (0.9)	997 (90.1)				47 (4.7)	959 (95.3)	Ref	Ref	
GDM										
Yes	0 (0.0)	128 (100)	–	–	–	2 (1.6)	126 (98.4)	0.30	(0.07–1.26)	0.099
No	9 (0.9)	942 (90.1)				48 (5.0)	903 (95.0)	Ref	Ref	
BV in the early stage of pregnancy										
Yes	0 (0.0)	74 (100)	–	–	–	3 (4.1)	71 (95.9)	0.86	(0.26–2.84)	0.806
No	9 (0.9)	996 (90.1)				47 (4.7)	958 (95.3)	Ref	Ref	
GBS-positive in the early stage of pregnancy										
Yes	4 (6.6)	57 (93.4)	14.22	(3.72–54.38)	< 0.001*	6 (9.8)	55 (90.2)	2.42	(1.01–5.91)	0.045*
No	5 (0.5)	1013 (99.5)	Ref	Ref		44 (4.3)	974 (95.7)	Ref	Ref	
BV during the 3rd trimester										
Yes		–	–	–		2 (3.6)	53 (96.4)	1.24	(0.29–5.31)	0.777
No	–				–	29 (3.0)	949 (97.0)	Ref	Ref	
GBS-positive during the 3rd trimester										
Yes	–	–	–	–	–	5 (5.1)	93 (94.9)	1.88	(0.71–5.01)	0.207
No						26 (2.8)	909 (97.2)	Ref	Ref	

*P*-value for bivariable regression analysis

*OR* Odds ratio, *CI* Confidence interval, *BMI* Body mass index, *GDM* Gestational diabetes mellitus, *BV* Bacterial vaginosis, *GBS* Group B Streptococcus

*Statistically significant

**Table 4:** Results from multivariable logistic regression analysis of factors associated with preterm birth

	Preterm birth < 34 weeks			Preterm birth < 37 weeks		
	Odds ratio	95% CI	*p*-value	Odds ratio	95% CI	*p*-value
Nullipara	0.30	(0.07–1.26)	0.298	–	–	–
Diabetes mellitus	8.06	(1.36–47.78)	0.022*	–	–	–
GBS-positive in the early stage of pregnancy	15.17	(3.73–61.74)	< 0.001*	2.42	(1.01–5.91)	0.045*

Variables were included in a logistic regression model, using backward elimination method to evaluate the independent association between variables and preterm birth

*CI* Confidence interval, *BV* Bacterial vaginosis, *GBS* Group B Streptococcus

*Statistically significant

## Discussion

A previous retrospective cohort study reported that GBS detection from 26 to 28 weeks of gestation was a risk factor for PTB before 37 weeks of gestation (OR: 2.24) [12]. Furthermore, our findings suggested that GBS colonization around 14 weeks of gestation was associated with PTB before 37 weeks of gestation (OR: 2.42, *p* = 0.045) as well as for PTB before 34 weeks of gestation (OR: 15.17, *p* < 0.001). To the best of our knowledge, only one report has discussed the relationship between vaginal culture during the 1st trimester and PTB [13]. Although the report showed no significant difference in the prevalence of PTB between the GBS-positive (*n* = 10) and GBS-negative (*n* = 211) groups, the number of cases analyzed was relatively less. In addition to PTB, the incidence of PROM was also relatively high in the GBS-positive group, although there was no significant difference. Our study revealed that diabetes is also one of the factors associated with PTB, as reported in previous reports [1]. One case-control study concluded that vaginal GBS colonization at 28–36 weeks of gestation was discriminant factors for pPROM according to stepwise discriminant analysis [14]. Our study yielded results consistent with this report, but a larger sample would be needed to demonstrate this association. Similarly, multiparity is reported as a risk of PTB [15], and our multivariable logistic regression analysis suggests that nulliparity was associated with decreased odds of PTB < 34 weeks of gestation, although there is no significant difference.

In the present study, we observed a relatively low rate of GBS detection (5.7%) in the early stage of pregnancy, whereas a previous study reported varying colonization rates from 5 to 49% [12]. One possible reason for the low detection rate in our study can be that the diagnosis of GBS is based on a rapid test. Assuming that our data revealed high colonization, such a result would support an association between high vaginal colonization in the early stage of pregnancy and PTB. Some researchers recommend performing the polymerase chain reaction (PCR) assay in addition to conventional culture method to prevent false negative and for the use of appropriate prophylactic antibiotics [16, 17], but conventional culture method rather than PCR is reported to be related with neonatal GBS infection itself [18]. No PCR was performed at our hospital, but none of the patients included in this study developed neonatal GBS disease. Early-onset GBS disease has become relatively uncommon in recent years due to many guidelines recommending continued efforts for preventing neonatal GBS disease [19]. Although the prevention of neonatal GBS infection is important, the higher prevalence of PTB suggests the need to focus on PTB during maternal GBS infection.

Our study has several strengths. First, our study indicates that patients at high risk for PTB can be extracted by a simple method using conventional culture method rather than PCR. It allows any facilities including local primary facilities to easily recognize high risk pregnant women. BV is also a known risk factor for PTB, but Nugent score, a diagnostic criteria, has the disadvantage of being laborious and difficult to reproduce [20], which hampers universal use. Second, this study has an important major, benefit of a large sample size. Many studies aimed at determining factors associated with PTB are limited by sample size, and this study addresses that. But our study has several limitations. First, the lack of data about sexually transmitted infections (STIs), which is reported to be a risk factor for PTB in the context of vaginal microbiota [21]. Second, the lack of performing GBS serotyping. Some studies emphasize the importance of serotyping of GBS because it is suggested that GBS has different clinical features depending on its serotype [22, 23]. Third is the lack of placental pathology that could have provided evidence to support the relationship between GBS infection and chorioamnionitis and placental membrane inflammation. Fourth, identifying the risk of PTB before 34 weeks of gestation is clinically valuable, but the number of cases is not large enough to obtain highly confident results about it. The multivariable regression analyses of identifying the risks of PTB < 34 weeks of gestation showed large 95% confident intervals, which are due to a small number of patients with PTB < 34 weeks of gestation. However, it is noteworthy that 4 of the 9 cases of PTB < 34 weeks of gestation were GBS positive in early stage of pregnancy.

Given that the present study examined the relationship between GBS detection in the early stage of pregnancy and delivery outcomes, the presence or absence of symptoms or treatment was not considered. Accordingly, to further investigate the association between GBS infection and PTB, additional research, including pathological evaluation or GBS serotyping or treatment effects, is imperative. Nevertheless, we believe that the present study could serve as the basis for further research.

## Conclusion

The present study found that vaginal GBS colonization in the early stage of pregnancy was associated with PTB. Future studies that consider treatment effects are necessary to translate to the clinical setting. Nevertheless, our results suggest that early prenatal screening for vaginal GBS colonization can be advantageous, given that it could help identify pregnant women at risk for PTB.

## Supplementary Information

**Additional file 1: Table S1.** Nugent score. **Table S2.** Association between BV and GBS detection during the 3rd trimester.

## Data Availability

The data that support the findings of this study are available from the corresponding author, ST, upon reasonable request.

## Abbreviations

PTB: Preterm birth
BV: Bacterial vaginosis
GBS: Group B streptococcus
PROM: Premature rupture of membranes
OR: Odds ratio
95%CI: 95% confident interval
PCR: Polymerase chain reaction
STIs: Sexual transmitted infections
