# Supplementary material for: Relationship between vaginal group B streptococcus colonization in the early stage of pregnancy and preterm birth: a retrospective cohort study
Source: BMC Pregnancy Childbirth. 2021 Feb 16;21:141. doi: 10.1186/s12884-021-03624-9 (PMC7888155; doi:10.1186/s12884-021-03624-9)
Supplement: Supplementary file 1 — Additional file 1: Table S1. Nugent score. Table S2. Association between BV and GBS detection during the 3rd trimester. [file 12884_2021_3624_MOESM1_ESM.docx]

**Supplementary data**

**Table S1. Nugent score.**

| **Score I** | **Score II** | **Score III** |
| --- | --- | --- |
| ***Lactobacillus***: Gram-positive rods/HPF | ***Gardnerella***: Small Gram-negative variable rods/HPF  ***Bacteroides***: Small Gram variable rods/HPF | ***Mobiluncus***: Curved Gram variable rods/HPF |
| 0: >30  1: 5–30  2: 1–4  3: <1  4: 0 | 0: 0  1: <1  2: 1–4  3: 5–30  4: >30 | 0: 0  1: <5  2: >5 |

HPF, High power field. Total score = I + II + III. 0–3, Negative for bacterial vaginosis; 4–6, Intermediate; 7–10, Bacterial vaginosis.

Table S2. Association between BV and GBS detection during the 3^rd^ trimester.

|  | GBS in early stage of pregnancy | | |
| --- | --- | --- | --- |
|  | Positive | Negative | p-value |
|  | n = 61 | n = 1,018 |  |
| BV in the early stage of pregnancy | 2 (3.3) | 72 (7.1) | 0.429 |
| BV during the 3^rd^ trimester | 5 (8.2) | 50 (4.9) | 0.221 |
| GBS-positive during the 3^rd^ trimester | 31 (50.1) | 67 (6.6) | <0.001* |

GBS, Group B Streptococcus; BV, Bacterial vaginosis. Data are presented as n (%). *Statistically significant
